# An Enquiry Concerning the Diseases and Functions of the Brain, the Spinal Cord, and the Nerves

**Published:** 1841-10

**Authors:** 


Art. XI.—*An Enquiry concerning the Diseases and Functions of the Brain, the Spinal Cord, and the Nerves.* By Amariah Brigham, m.d. —*New York,* 1840. 12mo, pp. 327.

We have had more than one occasion to express our high appreciation of the beneficial tendency of Dr. Brigham's publications, and the unpretending little treatise before us does not fall short of its predecessors in its character of practical utility :

"Its object," says the author in his preface, "is to call the attention of those practitioners of medicine into whose hands it falls, to the importance of the nervous system, and to persuade them to embrace every opportunity that is presented for studying its functions and diseases. For this purpose," he continues, "I have endeavoured to give a partial summary of what is now known respecting this system. I have collected a large number of cases explanatory of its diseases and functions, cases that are scattered through many volumes, to which I have added a considerable number that have fallen under my own observation, and have thus sought to indicate the way that this system should be studied, in order to increase our knowledge of its functions and our means of remedying its diseases. In the second part, I have briefly treated of a number of diseases, the pathology of which is not yet settled. I have not sought to give full accounts of these, but to direct attention to a few important circumstances, and such as require further investigation."

The first part of the volume is occupied by a general enquiry into the functions of the nervous system, as elucidated by its anatomy, by experiment, and by observation of its natural and morbid actions. Though this does not comprehend the details of the latest researches, there are no general views that can claim a fixed rank in neurology, which are not embodied in it; and the whole may be read with great profit by those who have not already given express attention to the subject. Dr. Brigham is evidently a partisan of no system, but in the true spirit of eclectic philosophy is ready to receive truth from whatever quarter she may present herself. The inductions on which he ventures are expressed with much caution; and there is throughout a complete avoidance of that dogmatic spirit, which, though more absurd when applied to medicine than to almost any other subject, figures perhaps more frequently in the pages of our literature than in that of any other branch of science.

The second part treats of the special diseases of the brain and spinal cord; and here the young practitioner will find much sound information, derived from the experience of a judicious physician, conveyed in a simple and attractive form. It seems to us that Dr. Brigham has most successfully attained the object he proposed to himself, and we cannot doubt that the influence of his little work will be highly beneficial wherever it receives due attention.

